# Effects of an adapted physical activity program in a group of elderly subjects with flexed posture: clinical and instrumental assessment

**DOI:** 10.1186/1743-0003-5-32

**Published:** 2008-11-25

**Authors:** Maria Grazia Benedetti, Lisa Berti, Chiara Presti, Antonio Frizziero, Sandro Giannini

**Affiliations:** 1 Movement Analysis Laboratory, Rizzoli Orthopaedic Institute, via di Barbiano 1/10, 40136 Bologna, Italy

## Abstract

**Background:**

Flexed posture commonly increases with age and is related to musculoskeletal impairment and reduced physical performance. The purpose of this clinical study was to systematically compare the effects of a physical activity program that specifically address the flexed posture that marks a certain percentage of elderly individuals with a non specific exercise program for 3 months.

**Methods:**

Participants were randomly divided into two groups: one followed an Adapted Physical Activity program for flexed posture and the other one completed a non-specific physical activity protocol for the elderly. A multidimensional clinical assessment was performed at baseline and at 3 months including anthropometric data, clinical profile, measures of musculoskeletal impairment and disability. The instrumental assessment of posture was realized using a stereophotogrammetric system and a specific biomechanical model designed to describe the reciprocal position of the body segments on the sagittal plane in a upright posture.

**Results:**

The Adapted Physical Activity program determined a significant improvement in several key parameters of the multidimensional assessment in comparison to the non-specific protocol: decreased occiput-to-wall distance, greater lower limb range of motion, better flexibility of pectoralis, hamstrings and hip flexor muscles, increased spine extensor muscles strength. Stereophotogrammetric analysis confirmed a reduced protrusion of the head and revealed a reduction in compensative postural adaptations to flexed posture characterized by knee flexion and ankle dorsiflexion in the participants of the specific program.

**Conclusion:**

The Adapted Physical Activity program for flexed posture significantly improved postural alignment and musculoskeletal impairment of the elderly. The stereophotogrammetric evaluation of posture was useful to measure the global postural alignment and especially to analyse the possible compensatory strategies at lower limbs in flexed posture.

## Background

The aging process modifies normal postural alignment, and flexed posture commonly increases with age. Thoracic kyphosis and protrusion of the head, and in more severe cases, knee flexion, characterize flexed posture [1-5].

Although the precise etiology of flexed posture (FP) is unknown, its pathophysiology in the elderly is most likely multifactorial and can be associated with low bone mineral density and consequently vertebral fractures, and also degenerative alterations of intervertebral disks [2,6-12]. It is also related to musculoskeletal and neuromuscular impairments [13-15]: deficit in spinal extensor muscle strength and shoulder and hip range of motion decreasing have been correlated with flexed posture [1,14-18]. Increased flexed posture has been associated with less independence when performing activities of daily living and reduced physical performance, such as impaired balance, reduced postural control and slower walking, as well as risk of falling [1,13,14,19-26]. Moreover kyphosis and compensative cervical and lumbar spine hyperlordosis can cause pain due to ligaments and muscles impairment [11,22].

There is no standard approach to measure flexed posture and classification methods are very complex [27]. Clinical assessments present the advantages of simplicity, low-cost and wide application, such as the occiput-to-wall distance [1] that classifies the severity of flexed posture. On the other hand, instrumental evaluation enables quantitative analysis of the global body alignment in flexed posture patients and especially the compensatory axial alterations at the head and lower limbs. In the literature, several studies have reported the use of goniometers, inclinometers [28,29], electrogoniometers [30], stadiometers [31-33] and photometric techniques [29,34-36]. The more recent stereophotogrammetric systems [37-39] seem to realize more reliable and valid evaluations of postural alignment analysis.

A sedentary life style is supposed to play a fundamental role in developing a flexed posture and there is evidence in literature that appropriate physical activity programs can correct this attitude [1].

There are few studies that investigate methods to improve flexed posture, and especially the relationship with the multiple impairments associated to flexed posture [17,40,41]. Sinaki [14,16,42-47] focused most research on the correlation among osteoporosis, kyphosis and back extensors strength and defined an exercise program based on isometric back-extensor strengthening and proprioceptive postural retraining to contain flexed posture [17,48-50]. In different studies, the same author analyzed the role of the physical activity program in improving balance, gait and quality of life and the consequences in reducing back pain, risk of falls and vertebral fractures [17,46,49-51]. The efficacy of these protocols seems to correlate to exercise specificity, but we failed to find sufficient randomized controlled trials in the literature that compare different programs.

Based on Sinaki's experience, we hypothesized that an Adapted Physical Activity (APA) program with specific exercises for flexed posture would improve postural alignment and physical performance in a more effective way than a non-specific physical activity protocol for the elderly. The purpose of this clinical study was to systematically compare the effects of a physical activity program that specifically address the flexed posture that marks a certain percentage of elderly individuals with a non specific exercise program for 3 months. A clinical multidimensional assessment [1], including evaluation of musculoskeletal impairment, motor function, and disability, and an instrumental assessment of global postural alignment were used as measures of outcome.

## Methods

### Subjects

The study included elderly subjects aged over 65 years with flexed posture. Fifty-one participants were recruited from a Senior Club and provided written informed consent to take part in the study. After multidimensional clinical assessment seventeen subjects were not enrolled because they had one of the following exclusion criteria: central nervous system disorders, secondary osteoporosis, postural hypotension, disabling blindness or deafness, known malignant neoplasia, history of known vertebral fractures, obesity with Body Mass Index (BMI) >30, Mini-Mental State Examination (MMS) [52] >23, New York Heart Association (NYHA) classification >1, Short Physical Performance Battery (SPPB) [53,54] with 1 item = 1.

The 34 subjects included in the study, (28 women and 6 men) with a mean age of 70.9 years (S.D. 5.1), were randomly divided into two therapeutic groups: Group APA followed an Adapted Physical Activity (APA) program with specific exercises for flexed posture and Group NSPA followed a non-specific physical activity (NSPA) protocol for the elderly. In both groups exercises were performed 2 days a week for 1 hour and the program lasted for 3 months.

The participants that completed the two programs (at least 80% of sessions) were: 15 subjects in Group APA (12 females and 3 males) with a mean age of 71.5 (S.D. 4.3), weight 66.5 kg (S.D. 9.8), height 156.9 cm (S.D. 10.5) and BMI 27.22 (S.D. 4.5); and 13 subjects in Group NSPA (10 females and 3 males) with a mean age of 71.5 (S.D. 4.9), weight 72.7 kg (S.D. 11.1), height 159.5 (S.D. 8.7) and BMI 28.79 (S.D. 5.2).

The research protocol was approved by the Rizzoli Orthopedic Institute Ethics Committee.

### Intervention

The exercises proposed for the APA group were aimed at improving flexibility at pelvic and shoulder girdle, and at strengthening back extensor muscles fighting the attitude to flexed posture. Most of exercises for back strengthening were selected among those proposed by Sinaki [49,51,55-57]. The set of exercises was discussed with the two physical trainers in charge for the groups management, in order to select exercises focused on specific impairment. We know in fact that both a deficit in spinal extensor muscle strength and reduction of flexibility at shoulders and hips have been correlated with flexed posture.

In both groups exercise sessions began with 10 minutes' warm-up and ended with 10 minutes' cool-down.

1. In a sitting position with hands behind the head, deep-breathing-in exercise combined with pushing elbows backwards. Then back to the initial position (10 repetitions).

2. In a sitting position with slightly flexed elbows, deep-breathing-in exercise combined with shoulder extension and adduction, and neck extension. Then back to the initial position (10 repetitions).

3. In a sitting position with arms along the sides, deep-breathing-in exercise combined with shoulder elevation. Then back to the initial position (10 repetitions).

4. In a sitting position with hands on thighs, deep-breathing-in exercise combined with shoulder abduction rotating palms upwards (10 repetitions).

5. In a sitting position holding a stick in two hands, deep-breathing-in exercise combined with raising the stick (8 repetitions).

6. In a sitting position with arms along the sides, lateral bending of the trunk while trying to touch the floor with fingers from one side to the other (8 repetitions).

7. In a standing position in front of a wall, arms overhead wall slides combined with neck extension (8 repetitions).

8. In a standing position with back touching the wall, starting from 90° shoulders abduction and 90° elbows flexion, complete shoulder abduction and elbow extension bringing hands over head (8 repetitions).

9. In a standing position with forearms on table, alternate hip extension (10 repetitions).

10. Supine with hip and knee flexion, and feet on the floor, anterior pelvic tilt while strengthening abdominal and glutei muscles (10 repetitions).

The non-specific physical activity protocol for the elderly adopted in Group NSPA consisted of global posture exercises through a floor training with the use of exercise balls for increasing joint mobility, muscle strength and flexibility.

### Clinical assessment

A multidimensional clinical assessment [1] on each subject was performed, including anthropometric data (height, weight and BMI), clinical profile, and measures of musculoskeletal impairment, motor function, and disability.

The Comorbidity Severity Index of the Cumulative Illness Rating Scale [58] was adopted to evaluate physical health status. The level of pain was measured using visual-analog scales [59] in: neck, thoracic spine and lumbar spine. We used: the Mini-Mental State Examination (MMS) [52] for cognitive status, the Geriatric Depression Scale (GDS) [60] for evaluating depression and the Multidimensional Fatigue Inventory (MFI) [61] for measuring fatigue.

Goniometry measurements were used to record range of motion (ROM) in the hips, knees, and ankles bilaterally, obtaining the following data: hip flexion, hip extension, hip adduction, hip abduction, hip internal rotation, hip external rotation, knee flexion, knee extension, ankle plantarflexion, and ankle dorsiflexion.

Muscular strength was measured by means of manual muscle testing (MMT) [62] in the following groups of muscles: spine extensors, abdominal muscles, abductors, adductors, extensors and flexors of the hip, flexors and extensors of the knee, and dorsiflexors and plantarflexors of the ankle.

We used four previously reported tests [1] to evaluate lengthening capacity of pectoralis major, back extensors, hamstring muscles, and hip flexors. Motor function was explored by means of the Short Physical Performance Battery (SPPB) [53,54] including balance test, gait speed test and chair-stand test. Disability was assessed using self-report instruments: the Barthel Index [63] and the Nottingham Extended Activities of Daily Living (ADL) Index [64].

The clinical evaluation of flexed posture was performed by measuring the occiput-to-wall distance [1], while subjects stood with heels and back touching a wall. All the clinical evaluations were performed by the same blinded physiatrist for the physical activity program assignment.

### Instrumental assessment

The instrumental assessment of posture was realized using a stereophotogrammetric system VICON 612 (Vicon Motion Systems, Oxford, UK) with 8 cameras (resolution 1.3 Megapixel, 100 Hz).

Twenty-seven reflective markers were placed on the subjects at the following anatomical landmarks of head, trunk, pelvis, thigh, shank, foot:

Head: glabella, right temporomandibular joint, left temporomandibular joint

Trunk: right acromion, left acromion, spinous process of 7^th ^cervical vertebrae (C7), medial point between the two spines of the scapula

Pelvis: right anterior superior iliac spine, left anterior superior iliac spine, right posterior superior iliac spine, left posterior superior iliac spine

Thigh: right greater trochanter, left greater trochanter, right lateral epicondyle, left lateral epicondyle

Shank: right tibial tuberosity, left tibial tuberosity, right head of the fibula, left head of the fibula, right lateral malleolus, left lateral malleolus

Foot: right calcaneus, left calcaneus, right first metatarsal head, left first metatarsal head, right fifth metatarsal head, left fifth metatarsal head.

During posture analysis, in order to relate the displacement of the marker arrays to the position of the 3D underlying bones, the Total 3D Gait was used [65]. The protocol developed for kinematic analysis of posture was designed at Rizzoli Orthopedic Institute and based on the Cast Protocol [65,66]. For the anatomical reconstruction of body segment motion, the following anatomical landmarks were calibrated: occipital protuberance, spinous process of 5^th ^lumbar vertebrae (L5), right medial epicondyle, left medial epicondyle, right medial malleolus, left medial malleolus, right second metatarsal head, and left second metatarsal head.

**Figure 1:**
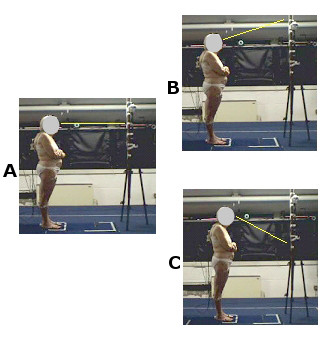
**Experimental set-up**. The subjects stands in an upright position staring at a target at eye level (static) (fig. 1A), 30% higher (extension) (fig. 1B) and 30% lower (flexion) (fig. 1C) than eye level.

The subjects stood in an upright position, with arms crossed and feet parallel (Fig. 1). For each subject 3 posture assessments, of 10 seconds' duration, were performed. For the first registration, the subjects were asked to stare at a fixed point one meter from away, at eye level (static) (Fig. 1A). For the second registration the participants were asked to stare at a visual target 30% higher (extension) (Fig. 1B) than their eye level. For the third registration the participants were asked to stare at a visual target 30% lower (flexion) (Fig. 1C) than their eye level. Three repetitions for each condition were registered for each subject.

**Figure 2:**
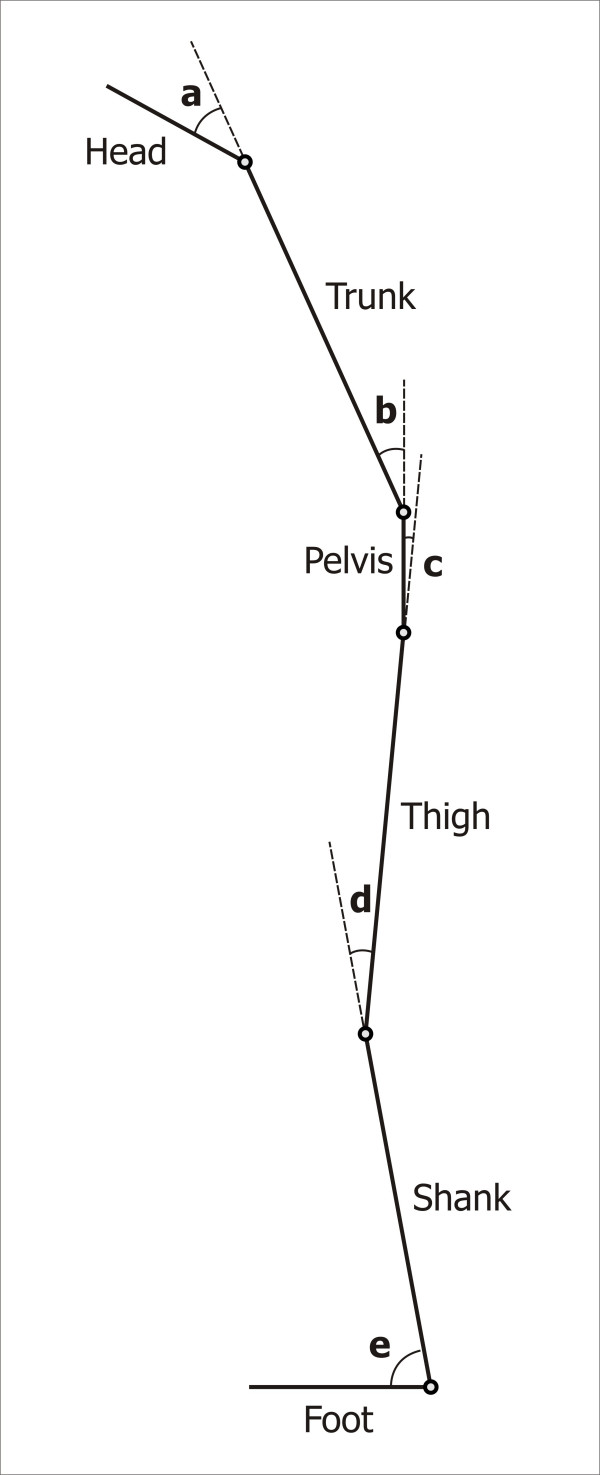
**Postural model**. The posture model describes the reciprocal position of the body segments with the following angles on the sagittal plane: head protrusion (a), trunk flexion (b), hip flexion (c), knee flexion (d), ankle dorsiflexion (e).

A specific posture model was constructed to describe the reciprocal position of the body segment with 8 angles on the sagittal plane (Fig. 2). These angles were defined as follows:

- head protrusion: the supplementary angle to the angle between head and trunk

- trunk flexion: the supplementary angle to the angle between trunk and pelvis

- right and left hip flexion: the supplementary angle to the angle between pelvis and femur

- right and left knee flexion: the supplementary angle to the angle between femur and shank

- right and left ankle dorsiflexion: the angle between shank and foot

### Statistical analysis

Continuous data were summarised in terms of means and standard deviation of the mean. The differences between baseline and 3 months follow up were investigated by the paired T-Test when the variances were homogenous and the Wilcoxon test when the variances were not homogeneous. All the analysis were considered significant for p < 0.05. Statistical Analysis was carried out by SPSS 15.0.

## Results

### Clinical assessment

Comparing the multidimensional clinical assessment performed at baseline and at 3 months, we noticed a statistically significant improvement of the occiput-to-wall distance (Table 1) only in Group APA.

**Table 1:** Clinical assessment in Group APA (Adapted Physical Activity) and in Group NSPA (Non-Specific Physical Activity) at baseline and 3 months.

Group APA						Group NSPA				
	baseline		after 3 months		p	baseline		after 3 months		p
				
	mean	SD	mean	SD		mean	SD	Mean	SD	

Occiput-to wall distance	**7.42**	1.96	**6.00**	1.93	0.001	8.48	2.63	7.88	2.28	ns

Pain score										
Lumbar spine	**4.97**	2.43	**2.73**	2.66	0.01	**4.83**	2.40	**1.85**	1.95	*0.003

ROM										
R hip extension	**12.60**	5.32	**20.40**	7.13	0.002	14.62	7.40	16.31	4.07	ns
L hip extension	**12.20**	5.59	**20.07**	6.66	0.003	13.23	7.47	16.15	3.71	ns
R knee flexion	**91.13**	1.36	**137.47**	6.64	*0.002	**98.54**	1.29	**136.00**	5.35	0.008
L knee flexion	**92.27**	3.83	**138.07**	4.83	*0.003	**98.46**	2.70	**137.31**	5.10	0.009
R knee extension	**3.00**	3.56	**0.00**	0.00	*0.01	**2.85**	4.56	**0.00**	0.00	*0.04
L knee extension	**3.27**	3.32	**0.00**	0.00	*0.008	**2.08**	2.87	**0.00**	0.00	*0.04
R ankle dorsiflexion	**4.47**	6.06	**12.27**	9.66	*0.002	6.69	4.75	8.46	2.25	ns
L ankle dorsiflexion	**4.53**	8.09	**9.53**	4.88	*0.012	6.00	3.76	7.85	3.80	ns

Flexibility										
R pectoralis major, cm	**9.26**	4.30	**7.40**	3.28	0.018	10.36	8.46	8.76	5.64	ns
L pectoralis major, cm	**9.20**	4.01	**7.03**	3,57	0.015	9.53	6.10	9.15	5.17	ns
R hamstrings, degrees	**70.13**	12.31	**78.20**	12.09	0.017	**70.54**	11.23	**77.00**	14.52	0.04
L harmstrings, degrees	**68.07**	12.77	**78.87**	13.45	0.011	**70.31**	10.62	**78.54**	8.02	0.052
R hip flexors, cm	**2.33**	1.79	**1.57**	1.33	0.079	2.12	1.64	1.81	1.50	ns
L hip flexors, cm	**2.50**	1.93	**1.20**	0.88	0.003	2.02	1.10	1.38	1.19	ns

Muscle strength										
Spine extensors	**4.21**	0.89	**4.86**	0.36	0.013	4.31	1.18	4.69	0.85	ns
Abdominals	**3.80**	1.52	**4.80**	0.41	*0.017	3.54	1.45	4.62	0.65	ns

p calculated with T-test, *p calculated with Wilcoxon test, ns = non-significant

Only parameters with a statistically significant difference between baseline and 3 months in almost one group were expressed.

Furthermore, we observed a greater improvement in lower limb range of motion in Group APA compared to Group NSPA; in the first group, many parameters increased (hip extension, knee flexion and extension and ankle dorsiflexion), whereas in the second one only knee flexion and extension increased (Table 1).

Considering muscular lengthening capacity, Group APA showed an improvement in three of the four flexibility tests regarding pectoralis major, hamstrings and hip flexors. Conversely, in Group 2 only the hamstrings flexibility increased (Table 1).

In both groups improving in abdominal muscles strength was evident after the exercise programs, but spine extensors significantly increased their strength only in Group APA (Table 1).

Short Physical Performance Battery scores improved in both groups: in Group NSPA with a statistically significant difference, whereas the difference was close to significance (p = 0.07) in Group APA (Table 2).

**Table 2:** Comprehensive geriatric assessment in Group APA (Adapted Physical Activity) and in Group NSPA (Non-Specific Physical Activity) at baseline and 3 months.

Group APA						Group NSPA				
	baseline		after 3 months		p	baseline		After 3 months		p
				
	mean	SD	mean	SD		Mean	SD	mean	SD	

Mini-Mental State Examination *(max 30)*	28.93	1.90	28.93	1.79	ns	28.69	1.97	28.62	2.02	ns

Geriatric Depression Scale *(0–30)*	5.33	4.73	4.40	5.22	ns	4.08	2.53	3.62	2.59	ns

Multidimensionall Fatigue Inventory (20–100)	38.73	13.78	34.53	15.39	ns	37.62	10.02	36.31	8.60	ns

Short Physical Performance Battery *(max 12)*	**10.33**	1.17	**11.07**	0.79	0.07	**9.38**	1.04	**10.46**	1.61	0.028

Barthel Index *(max 100)*	98.33	2.44	99.00	2.07	ns	98.85	2.19	100.00	0.00	ns

Nottingham Extended ADL Index *(max 22)*	20.80	1.20	21.00	1.00	ns	20.92	1.18	21.00	1.15	ns

p calculated with T-test, *p calculated with Wilcoxon test,, ns = non-significant

After exercise, there was no statistically significant difference in the two groups in the following scores: Mini-Mental State Examination, Geriatric Depression Scale, Multidimensional Fatigue Inventory, Barthel Index and Nottingham Extended Activities of Daily Living Index (Table 2).

The level of pain, evaluated by visual-analog scales, was only reduced in lumbar spine by both activity programs in a statistically significant way.

### Instrumental assessment

Regarding posture instrumental assessment, we compared the angles obtained at baseline and at 3 months.

In the standing position with visual target at eye level (static), we found, after exercise, a reduction in flexed posture characterized by diminishing protrusion of the head and ankle dorsiflexion in Group APA (Table 3). Conversely, Group NSPA did not show any statistically significant differences in posture angles after the activity program (Table 3).

**Table 3:** Instrumental assessment in Group APA (Adapted Physical Activity) and in Group NSPA (Non-Specific Physical Activity) at baseline and 3 months.

Group APA						Group NSPA				
	baseline		after 3 months		p	baseline		after 3 months		p
				
	mean	SD	mean	SD		mean	SD	mean	SD	

STATIC										
head protrusion	**40.31**	8.79	**35.16**	9.60	0.04	39.81	10.85	40.51	9.29	ns
R ankle flexion	**80.74**	3.42	**83.11**	3.85	0.01	80.21	4.78	80.97	5.23	ns
L ankle flexion	**80.4**	4.21	**83.81**	3.94	0.01	81.65	5.26	83.23	5.05	ns

EXTENSION										
R knee flexion	**6.58**	2.67	**4.10**	2.41	<0.0005	**5.85**	3.55	**3.27**	1.95	0.01
L knee flexion	**6.41**	3.61	**3.87**	1.92	<0.0005	**6.29**	5.11	**3.87**	1.84	0.04

FLEXION										
head protrusion	**50.71**	4.86	**40.08**	7.02	<0.0005	49.89	7.11	46.96	5.36	ns
R ankle flexion	**80.14**	3.34	**83.45**	3.60	<0.0005	80.86	5.48	81.36	4.52	ns
L ankle flexion	**81.94**	3.86	**84.00**	3.60	0.04	82.16	3.59	82.85	5.13	ns

p calculated with T-test,, ns = non-significant

Only parameters with a statistically significant difference between baseline and 3 months in almost one group were expressed.

In the standing position with visual target at a higher lever (extension), both groups had a decrease in knee flexion at follow up, although in Group APA this difference was more significant (Table 3).

In the standing position with visual target at a lower lever (flexion), Group APA showed a statistically significant reduction of protrusion of the head and a reduction of ankle dorsiflexion after exercise (Table 3).

## Discussion

Considering the multifactorial pathophysiology of FP, we enrolled patients according to strict inclusion criteria to avoid confounding factors.

The multidimensional clinical assessment [1] was an excellent mean to characterize the elderly population because, as documented in literature [52-54,60,61,63,64], specific for studying the complex clinical profiles and impairments in the geriatric field.

Based on the well known concept that back extensors weakness is definitely recognized as a key element in the pathophysiology of FP [1,14,67], exercised for strengthening of back extensor muscles were included in the APA protocol. Other exercises in the APA protocol were addressed to the stretching of muscles of hip and shoulder flexors. Besides a hip flexor static contracture, common in the elderly [68], we have to consider in fact that retraction of hip flexors and of anterior muscles of shoulders and neck is an expected consequence of flexed posture.

The results support our primary hypothesis that a Physical Activity program adapted to the specific impairment is more effective in improving flexed posture in the elderly than a non-specific protocol. This effect is evident when considering the spine extensor muscle strength, significantly increased only in the APA group. Moreover, even if both exercise programs had positive effects on knee ROM and hamstrings flexibility, the specific APA protocol was more effective in those districts mainly involved in flexed posture [1,22,40,44], modifying ROM of hip joint and increasing the flexibility of hip flexor muscles and of the pectoralis major. The improvement of these measures only in the Group APA confirmed the specificity of the APA program in modifying soft tissue retraction associated to flexed posture.

As a global measure of outcome in quantifying flexed posture, it is relevant that the occiput-to-wall distance, considered a specific indicator of severity of FP [1], showed a statistically significant decrease only in the APA Group.

The instrumental assessment of posture allowed us to measure the global postural alignment and especially to analyse the possible compensatory strategies to FP. The experimental set up was quite simply and easily reproducible. Considering the complexity of classification methods for posture in the literature [27], we designed a specific biomechanical model based on five different angles on the sagittal plane. These angles, clinically meaningful, (head protrusion, trunk flexion, hip flexion, knee flexion, ankle dorsiflexion) referred to precise compensatory axial deviations to kyphosis, that are important components of FP [1,21]. The three different visual tests (static, extension, and flexion) were performed to study the postural adaptations during eye-level modifications; this analysis allowed us to define the impact that the axial deformity of FP has on daily living activities (ADL), simulating dynamic activities which could be performed during ADL. While any compensatory movement is expected in normal people when looking at the top or down due to normal postural alignment and adjustment ability, we hypothesized that people with flexed posture and reduced mobility at the level of upper spine would adopt possible compensation strategies, mainly bending the knees and increasing ankle dorsiflexion in order to control balance.

Even in the instrumental evaluation, the specific APA program provided more significant improvements in the control of postural alignment than the non-specific protocol. In detail, both in static and flexion positions we observed a reduction in the flexion of the head and dorsiflexion of the ankle. In the extension position, the two programs determined the same positive effects with the decrease in knee flexion.

The more aligned head position instrumentally measured in the static posture and in the "extension test" in the APA Group confirms the reduction of the "occiput to wall distance" clinically measured. Moreover instrumental postural assessment highlights the increased ankle dorsiflexion as a compensatory strategy adopted by patients in order to maintain balance before the physical activity period, which reduces after training. The recovery of a better alignment of head and spine after specific exercise reduces the need of a biomechanical compensation at the ankle. The same mechanism can be supposed for the reduction of knee flexion in the "flexion test". In this case the improvement was observed in both groups, even more evident in the APA Group.

General physical performance increased in both groups, as the SPPB scores demonstrated. This was probably due to the positive effects that exercises, in general, have on balance, gait and motor function in the elderly. Conversely, after analyzing the Barthel Index [64] and the Nottingham Extended Activities of Daily Living Index [65] we could not find any improvement in the disability of the elderly after exercise. The reason for this can be attributed to the high-level of independence of the elderly population before treatment with a possible ceiling effect of score systems used. The slight association with disability might be due to the use of effective compensatory strategies even in the presence of severe FP, as previously hypothesized [1]. This finding confirms the results of other authors who found a weak correlation between severity of FP and disability [1,4,21]. The measure of cognitive status [52], depression [60], and fatigue [61] did not reveal any statistically significant changes; these results were consistent with the high values found before exercise. All patients complained of lumbar pain, improved after both the physical activity programs. As back pain is probably related to abnormal stress of muscles and ligaments [11,22], it is reasonable that even a postural non-specific physical activity program can be effective in relieving this symptom.

The Adapted Physical Activity program was inspired by the well-documented Sinaki approach, based on the very important role of Spinal Proprioceptive Extension Exercise Dynamic (SPEED) program [51,55,56]. Sinaki amplified her own protocol with the use of a spinal weighted kypho-orthosis (WKO) to increase a patient's perception of spinal positioning [17,43,50]. We limited our program to the 10 selected exercises without using any orthosis.

The good results of the present trial are in agreement with findings of previous studies [14,17,40,41,44,48] that investigated methods to improve flexed posture, all based on back-extension strengthening exercises. However, it was not possible to make a precise comparison of previous findings due to the different measurement systems adopted. The peculiarity of our research consisted of the randomized controlled clinical trial and the instrumental quantitative analysis of flexed posture. A limitation is however related to the small sample size of eligible subjects due to strict criteria of inclusion and different number of subjects which concluded the study in the two groups. The larger benefit achieved with the APA program compared to the non-specific activity program essentially refers to the investigation herein presented as a preliminary study. Further development of the present research is required particularly to calibrate the optimal amount of exercise to be administered taking into account even individual impairment. As Sinaki [46] affirmed, an exercise program aimed at maintaining muscular strength and flexibility is characterized by the principle of reversibility, so discontinuation reverses the improvement to pre-exercise levels. For this reason, the results of this research may be useful in developing a long-term Adapted Physical Activity program for the elderly aimed at containing and preventing FP.

## Conclusion

The Physical Activity program adapted for people with flexed posture improved postural alignment and musculoskeletal impairment more effectively than a non-specific physical activity protocol. The increasing of back extensors strength, the increasing in the flexibility of pectoralis, hip flexors and hamstrings muscles correspond to the reduction of FP, as measured by means of the occiput-wall distance. The instrumental assessment, based on a clinically oriented, reliable biomechanical model, allowed to measure the global postural alignment in patients with FP before and after physical activity trials and especially to analyse the possible compensatory strategies at the head and lower limbs.

## Competing interests

The authors declare that they have no competing interests.

## Authors' contributions

MGB made substantial contributions to conception and design of the study, analysis and interpretation of data and she was involved in drafting the manuscript and revising it critically for relevant intellectual content. LB performed all the measurements and was involved in drafting the manuscript. CP and AF participated in data acquisition and analysis. SG gave final approval of the version to be published.
